# Nanobiotechnology-mediated regulation of reactive oxygen species homeostasis under heat and drought stress in plants

**DOI:** 10.3389/fpls.2024.1418515

**Published:** 2024-08-27

**Authors:** Linfeng Bao, Jiahao Liu, Tingyong Mao, Linbo Zhao, Desheng Wang, Yunlong Zhai

**Affiliations:** ^1^ College of Agriculture, Tarim University, Alar, China; ^2^ Key Laboratory of Tarim Oasis Agriculture, Ministry of Education, Tarim University, Alar, China

**Keywords:** drought stress, heat stress, plant nanobiotechnology, chloroplasts, mitochondria, apoplast, ROS homeostasis

## Abstract

Global warming causes heat and drought stress in plants, which affects crop production. In addition to osmotic stress and protein inactivation, reactive oxygen species (ROS) overaccumulation under heat and drought stress is a secondary stress that further impairs plant performance. Chloroplasts, mitochondria, peroxisomes, and apoplasts are the main ROS generation sites in heat- and drought-stressed plants. In this review, we summarize ROS generation and scavenging in heat- and drought-stressed plants and highlight the potential applications of plant nanobiotechnology for enhancing plant tolerance to these stresses.

## 1 Introduction

Water scarcity is a constraint on agriculture, and global warming is exacerbating the problem. Drought caused by high temperature has become one of the major abiotic stresses threatening crop yield globally (Lippmann et al., 2019; Dietz et al., 2021). The United Nations report shows, the global surface temperature over 2011–2020 was about 1.1°C higher than that over 1850–1900, and the land surface temperature was 0.71°C higher than the sea surface temperature (Calvin et al., 2023).According to a report released by the Food and Agriculture Organization of the United Nations (FAO) and World Meteorological Organization (WMO), the number of people threatened by food security worldwide has increased significantly from 25.3% in 2019 to 29.6% in 2022 (FAO, 2023), and the heat and drought in 2023 have affected the food security of millions of people (Climate change indicators reached record levels in 2023: WMO, n.d). Lifting the constraints imposed on agriculture by high temperatures and drought can further unleash the potential of agricultural production to effectively respond to the food crisis.

Reactive oxygen species (ROS) in plant cells mainly include superoxide anion radical (O_2_^•−^), hydrogen peroxide (H_2_O_2_), hydroxyl radical (^•^OH), and singlet oxygen (^1^O_2_) (Dat et al., 2000; Halliwell, 2006; Mittler, 2017). Normally, ROS are key signaling molecules in plant cell cycle regulation and programmed death (Gapper and Dolan, 2006; Livanos et al., 2012; Petrov et al., 2015; Qi and Zhang, 2020), and they interact with signaling molecules such as salicylic acid, jasmonic acid, ethylene, and abscisic acid and are the upstream or downstream regulators of many metabolic pathways in plants (Kwak, 2003; Jammes et al., 2009; Denness et al., 2011; Steffens, 2014; Herrera-VÃ¡squez et al., 2015). The excessive accumulation of ROS under high temperature and drought stress can have toxic effects on plants (Suzuki and Mittler, 2006; Djanaguiraman et al., 2018b; Hussain et al., 2019). High levels of ROS can lead to DNA breakage as well as the inactivation of photosystem II (PSII) in the chloroplasts, thereby inhibiting photosynthesis (Kruk et al., 2005). Excessive ROS levels can impair plant growth and development, while maintaining ROS homeostasis is beneficial to plant growth (Dat et al., 2000; Mittler, 2017). The ROS in plant cells are mainly formed in the chloroplasts, mitochondria, peroxisome, and apoplasts. Plants also have antioxidant systems (Mittler et al., 2004; Impa et al., 2012), including both enzymatic and non-enzymatic systems (Hernández et al., 2012; Rajput et al., 2021), that protect cells from the excessive accumulation of ROS. The antioxidant enzyme system includes superoxide dismutase (SOD), peroxidase (POD), catalase (CAT), ascorbate peroxidase (APX), and glutathione peroxidase (GPX) (Alscher et al., 2002; Laxa et al., 2019)

Non-enzymatic systems include small organic molecules with antioxidant capabilities, such as polyphenols, carotenoids, vitamin C, glutathione, melatonin and polyamines (Hernández et al., 2012; Kolupaev et al., 2023). Maintaining ROS homeostasis can improve crop resistance to high temperature and drought stress (Awasthi et al., 2015; Verma et al., 2019).

In recent years, it has been reported that nanoparticles improve plant abiotic stresses tolerance. For example, iron oxide nanoparticles ameliorated the cadmium and salinity stresses in wheat plants (Manzoor et al., 2021). Application of ZnO nanoparticles could alleviate the low temperature damage on the early growth of rice (Mai et al., 2024). Emerging studies focuse on the use of plant nanobiotechnology to regulate plant ROS homeostasis to improve plant resistance to stress (Zhao et al., 2020; Liu et al., 2021a).To date, several nanomaterials, such as CeO_2_ (Khan et al., 2021), and Mn_3_O_4_ (Liu et al., 2023) were reported as an ROS scavenger in plant abiotic stress tolerance. For example, cerium oxide nanoparticles enable plants to withstand drought and saline-alkali stress (Djanaguiraman et al., 2018b; Liu et al., 2021). The high temperature tolerance of plants is improved with nano-selenium treatment (Djanaguiraman et al., 2018a). In addition, some nanomaterials can maintain ROS homeostasis and enhancing plants heat and drought tolerance (Wu et al., 2017).

Nanobiotechnology can effectively maintain ROS homeostasis and improve plant resistance to heat and drought stress. Therefore, in this paper, we presented a overview of how plants generate and scavenge ROS during heat and drought stress. Also show the role of nanobiotechnology in maintaining ROS homeostasis in crops, the underlying potential mechanism in enhancing crop resistance and ensuring sustainable agriculture.

## 2 Effect of heat and drought stress in plants

Drought and heat stress affect the plant morphology and physiological functions. For instance, both stresses retard the photosynthetic machinery and limit photosynthesis (Carmo-Silva et al., 2012), cause ROS over-accumulation (Das and Roychoudhury, 2014), and hinder plant growth and development (Yadav et al., 2022).

Combined heat and drought exacerbates the adverse effects on plants compared to individual stresses. Drought typically causes stomatal closure (Buckley, 2019), whereas heat stress results in stomatal opening (López et al., 2022). During concurrent heat and drought stress, drought can exacerbate thermal damage during heatwaves (Marchin et al., 2022). Both stresses lead to ROS over-accumulation. For example, it was reported that heat and drought stress cause ROS accumulation and suppress seed germination and growth in rice (Liu et al., 2019). Therefore, maintaining ROS homeostasis is a must to do physiological process in plants to ensure survival. This could be achieved through modulating the activity of ROS scavenging pathways and recruiting antioxidant enzyme activities. However, heat and drought significantly decrease the activities of antioxidant enzymes such as SOD, POD, and CAT (Hu et al., 2023), which is detrimental to maintaining ROS homeostasis in plants. Hence, investigating options to assist plants performance under harsh conditions it is particularly important task to explore.

## 3 ROS generation in plants

### 3.1 ROS in the mitochondria

**Figure 1:**
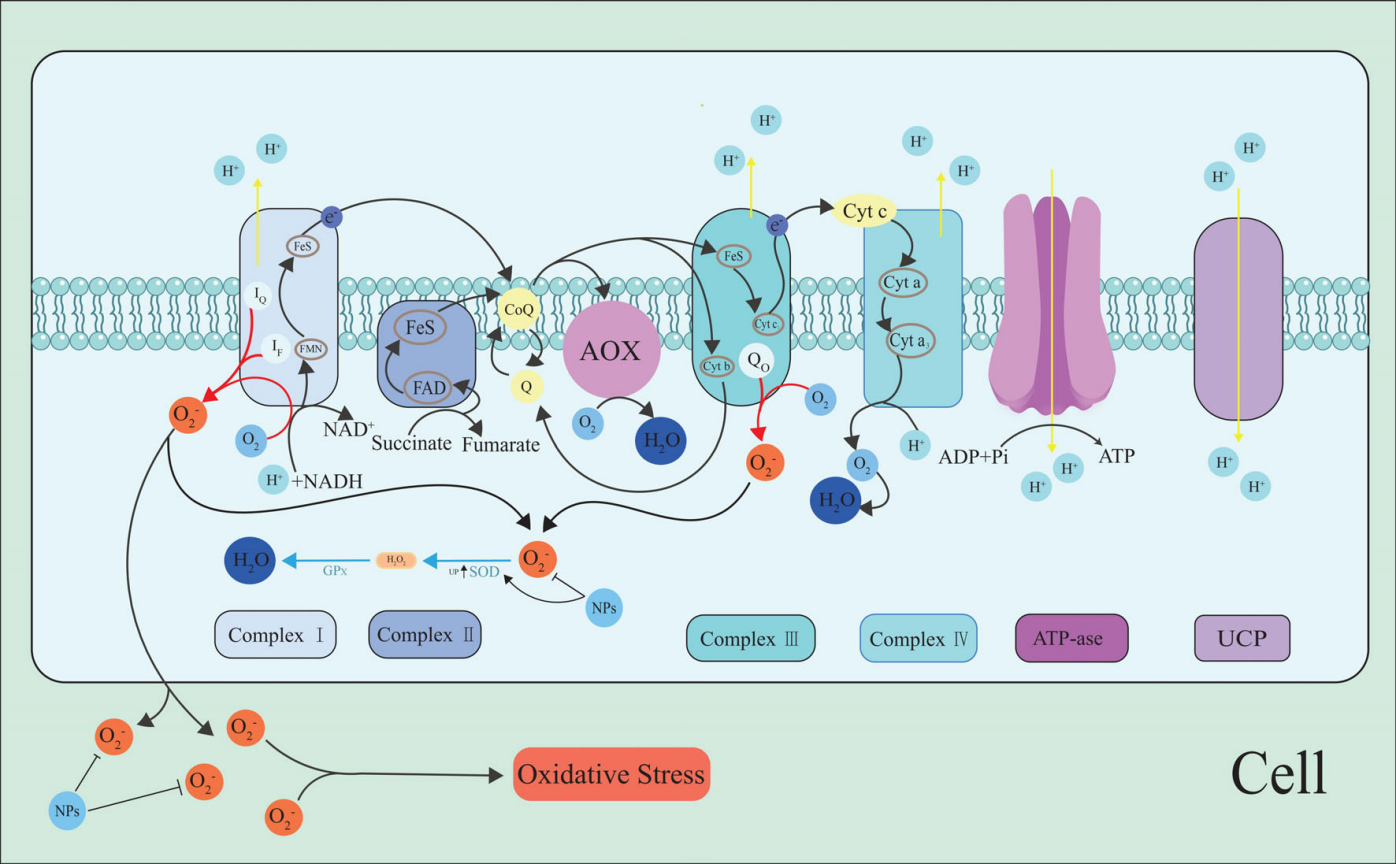
ROS generation along the RETC pathway. O_2_^•−^ is formed upon single-electron reduction of O_2_ (Chenna et al., 2022). Complex I and III in the RETC are the major sites of O_2_^•−^ production. UCP and AOX in RECT. The energy of the proton gradient drives the ATP synthesis of or can be consumed by UCPs. NPs inhibit the ROS generation and scavenge ROS, maintain cell ROS homeostasis. The black arrow indicates the electron pathway or ROS transfer, and the red arrows represent O_2_^•−^ generation.

The mitochondria are one of the ROS production sites. In plant cells, most of the ROS produced under light come from the chloroplasts or peroxisomes, but in non-green tissues or darkness, mitochondrial ROS production dominates (Navrot et al., 2007) (**Figure 1**). This production is an inevitable result of aerobic metabolism, and about 1–5% of the oxygen consumption of plants is due to H_2_O_2_ production (Černý et al., 2018). The main site of ROS formation in the mitochondria is the respiratory electron transport chain (RETC), which involves the reduction of oxygen to H_2_O. Different pathways are involved in this reduction process. Some enzyme systems, such as cytochrome c oxidase (COX), directly reduce oxygen, while others reduce oxygen in steps, i.e., oxygen accepts only one electron, resulting in the formation of O_2_^•−^. Nicotinamide adenine dinucleotide (NAD^+^) is an important coenzyme in RETC in the inner mitochondrial membrane. Also, it is an important electron acceptor in the RETC. NADH, ubiquinone oxidoreductase (Complex I), and the cytochrome C reductase complex (Complex III) is the main site of ROS production, Complex I mainly produces a small amount of O_2_^•−^, and Complex III produces O_2_^•−^ which is then converted to H_2_O_2_ (Gakière et al., 2018; Dourmap et al., 2020).

Mitochondrial damage caused by oxidative stress can trigger the release of metals in mitochondrial proteins (Mittler et al., 2004). In the presence of high levels of O_2_^•−^ and H_2_O_2_, the release of metals accelerates the reaction to generate ^•^OH, thus causing membrane damage through lipid peroxidation (Tan et al., 2010; Mittler, 2017; Suzuki, 2023). Some studies have shown that the content of Cu and Fe in the mitochondria is reduced by 40% after oxidative stress, which indicates that the complex containing these two metals in the electron transport chain on the mitochondrial membrane is damaged (Tan et al., 2010). This may lead to an increase in free ferrous ions, with H_2_O_2_ and Fe^2+^ forming highly toxic ^•^OH, which damages plants (Mittler, 2017). As these complexes are sensitive to abiotic stresses, their protection is important for maintaining ROS homeostasis.

Uncoupling protein (UCP) is a membrane albumen located in the inner membrane of the mitochondria that belongs to the mitochondrial anion operating family. One study found that the increased expression of UCP in the tobacco mitochondria may lead to greater tolerance of drought stress (Begcy et al., 2011). Further research on the mechanism of UCP in maintaining mitochondrial ROS homeostasis may unearth the great potential of plants in the face of abiotic stresses.

Alternation oxidase (AOX) is another mitochondrial protein involved in maintaining cellular metabolism and energy balance, and its role is even more important under stress (Kumari et al., 2019; Garmash, 2022).AOX reduces excess oxygen in mitochondria by providing additional electron consumption pathways, thereby reducing ROS production and maintaining ROS homeostasis (Maxwell et al., 1999; Analin et al., 2023).In addition, AOX plays an important role in ethylene-induced drought resistance and improves drought resistance in tomato seedlings by balancing ROS levels in autophagy production (Zhu et al., 2018).Under osmotic and temperature stress, AOX pathway regulation of ROS through redox couples related to malate valve and antioxidant system (Dinakar et al., 2016). A study found that a lack of AOX under drought stress led to an increase in cell damage and a decrease in the ability of tobacco plants to recover quickly once the water supply was restored (Wang and Vanlerberghe, 2013). In another study AOX metabolites alongside the activation of several AOX enzymes relieve impact of combined heat and salt stresses on tomato (Sousa et al., 2022).

### 3.2 ROS in the chloroplasts

**Figure 2:**
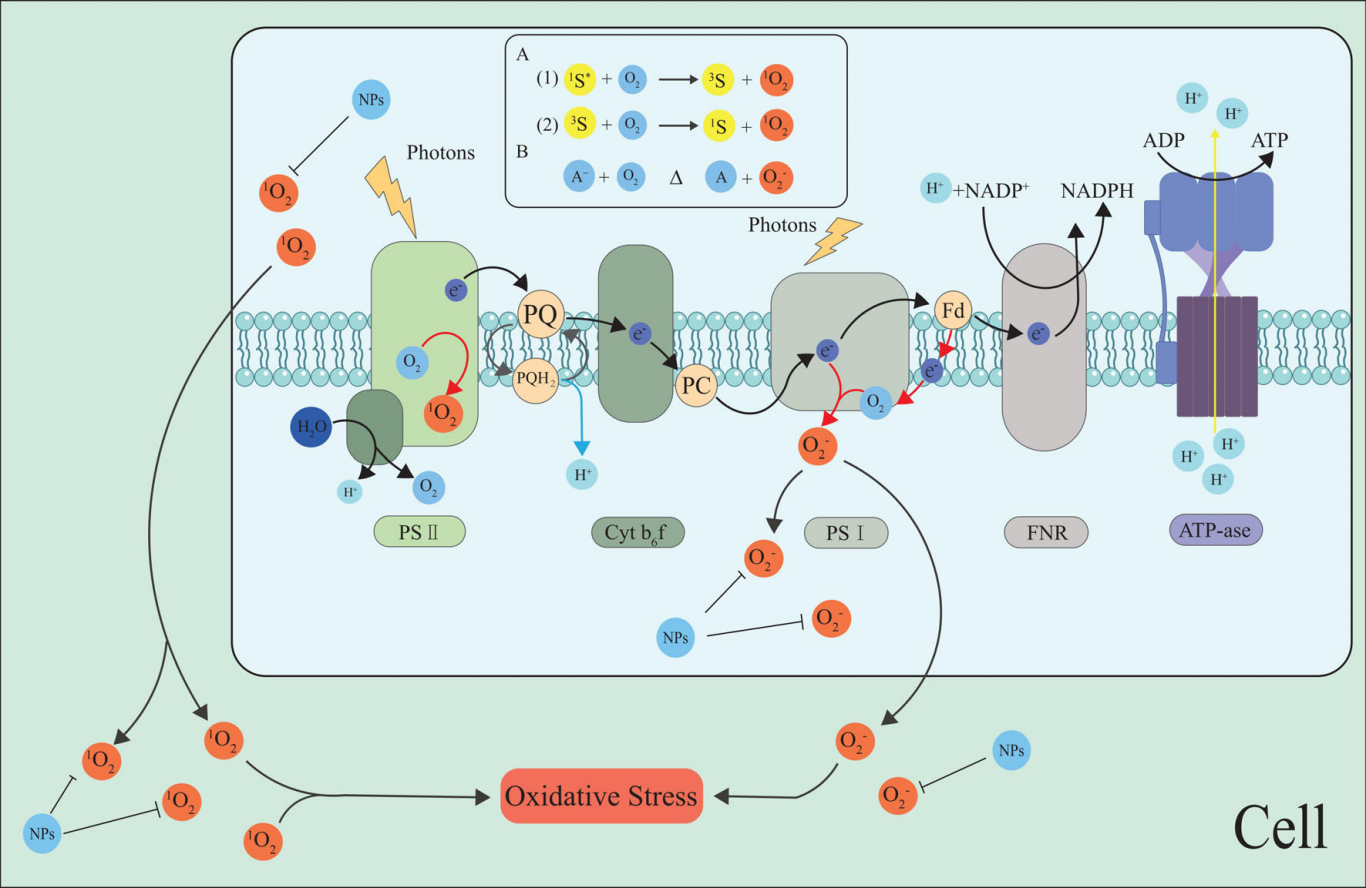
The generation of ROS in the photosynthetic electron transport chain. Photoexcitation of chlorophyll at PSII and PSI drives electron transport, but in the absence of acceptors, excitation may transfer to O_2_ at the PSII reaction center, forming a ^1^O_2_ and O_2_^•−.^ NPs inhibit the ROS generation and scavenge ROS, maintain cell ROS homeostasis. The most common mechanism of ^1^O_2_ generation is photosensitization, i.e., the reaction of O2 with a photoexcited sensitizer dye (S^*^). Produced by spin-conserved Reactions (A1) and (A2). O_2_^•−^ is mainly formed by the interaction of O_2_ with reducing compounds (A) with low redox potential (B) (Khorobrykh et al., 2020).The black arrow indicates the electron pathway or ROS transfer, and the red arrows represent O_2_^•−^ generation. Fd, ferredoxin; FNR, ferredoxin-NADP reductase; PC, plastocyanin; PQ, plastoquinone; and Cytb_6_f, Cytochromeb_6_fcomple.

**Figure 3:**
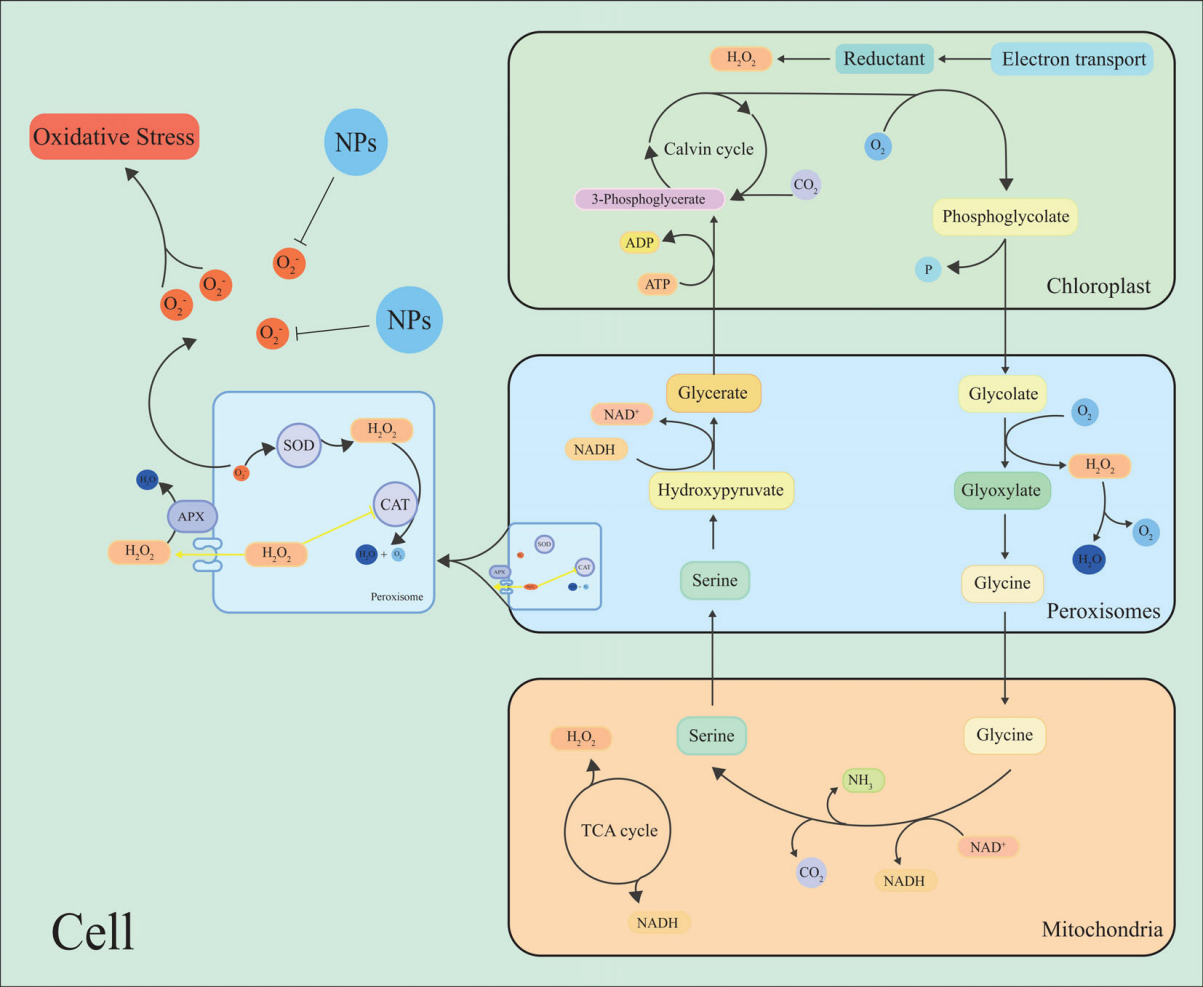
Simple model for the production or removal of H_2_O_2_ by plants through photorespiration and peroxisomal metabolism. During photorespiration, when oxalates are oxidized to glyoxylate by enzymes called glycolates, H_2_O_2_ is produced as a by-product. NPs inhibit the scavenge ROS, maintain cell ROS homeostasis.

As the main site of photosynthesis, chloroplasts are important organelles in plants, and the photosynthetic electron transport chain (PETC) on the thylakoid membrane is crucial for plant photosynthesis. Chloroplasts not only carry out photosynthesis but also sense and transmit stress signals under abiotic stresses such as high temperature and drought (Foyer and Shigeoka, 2011; Hu et al., 2020b; Somal et al., 2023). They are also the most important plant parts for producing ROS under stress. Photosystem I (PSI) and PSII in the PETC on the thylakoid membrane are the main sites for ROS production (Takahashi and Asada, 1988; Roach and Krieger-Liszkay, 2014) (**Figures 2**, **3**).

The Mehler reaction is the main pathway of ROS production in the chloroplasts. During plant stress, the Mehler reaction reduces O_2_ to O_2_^•−^ by PETC in PSI (Kozuleva et al., 2020a, Kozuleva et al., 2020b). PSII also produces ROS such as H_2_O_2_ (Pospíšil, 2009). Water-water cycling (WWC) is a highly efficient photoprotection strategy that can effectively scavenge O_2_^•−^ and H_2_O_2_ from the thylakoids to protect plants from oxidative damage (Asada, 1999, Asada, 2000; Rizhsky et al., 2003; Awad et al., 2015; Huang et al., 2019). Additional findings suggest that WWC enhancement at high temperatures protects PSI, while cyclic electron flow enhancement at low temperatures compensates for WWC deactivation, both of which together protect plants from ROS damage (Sun et al., 2020; Jiang et al., 2021). Maintaining chloroplast ROS homeostasis by regulating WWC may be an effective way to improve plant stress resistance.

Nicotinamide adenine dinucleotide phosphate (NADP) is one of the end electron acceptors in the electron transport chain of photosynthesis. The reduced nicotinamide adenine dinucleotide phosphate (NADPH) is an important reducing force in chlorophyll synthesis and the Calvin cycle. NAD kinases (NADK) are involved in many plant life activities, such as maintaining intracellular REDOX balance and responding to environmental stress. Studies have shown that NADK activity mediates response mechanisms to regulate PSI biosynthesis (Ji et al., 2022). Because NADP can provide the main reduction power for the ROS scavenging system (Mittler, 2002).In addition, NADK deficiency results in decreased ROS stress and drought tolerance in plants (Sun et al., 2017; Wang et al., 2020; Chaomurilege et al., 2023).

Non-photochemical Chlorophyll fluorescence quenching (NPQ) is a process that occurs in plants where excess absorbed light energy is converted into heat energy (Demmig-Adams et al., 2014). The relationship between NPQ and ROS homeostasis is complex. When the NPQ generation process is inhibited, such as by thermal inactivation of APX, the formation of ROS increases, which can lead to plant damage. It was found that heat treatment could significantly stimulate NPQ, while increasing the temperature almost completely inhibited NPQ, and was accompanied by the generation of ROS (Hideg et al., 2008). This indicates that the NPQ generation and ROS clearance processes are closely linked. According to Liu et al. (2022) the significantly higher non-photochemical quenching (NPQ) in bundle sheath chloroplasts compared to mesophyll chloroplasts under drought stress could be a primary reason for the lower accumulation of ROS in bundle sheath chloroplasts. Studying the relationship between NPQ and ROS homeostasis and the light damage resistance of chloroplasts is important for improving plant stress resistance.

### 3.3 ROS in the apoplasts

Apoplasts are composed of intercellular space, a cell wall, and an extracellular matrix, and they play an important role in the transport of water, mineral nutrients, and organic nutrients, as well as the production of signaling molecules in response to stress (Hoson, 1998; Sattelmacher, 2001; Koch et al., 2003; Qi et al., 2017). Although the level of ROS production in the apoplasts is much lower than that of the chloroplasts and mitochondria, as the site of ROS production, it is no less important to plants than the chloroplasts and mitochondria (Jubany-Mari et al., 2008; Barceló and Laura, 2009). It plays an important role in the plant perception of external stresses (Mittler, 2017; Mittler et al., 2022).

In the apoplasts, ROS are mainly produced by NADPH oxidase (Podgórska et al., 2017). Respiratory burst oxidase homologs (RBOHs) are the main sites of ROS production in apoplasts, and they transfer electrons from cytosolic NADPH to O_2_ and then produce O_2_^•−^ in apoplast bodies, following which they generate relatively stable H_2_O_2_ catalyzed by SOD and then enter the cell as a signaling molecule (Sagi and Fluhr, 2006; Marino et al., 2012; Hu et al., 2020a). NADPH acts as a fuel for RBOHs and provides sufficient substrate for ROS production when plants encounter stress (Wu et al., 2023a). ROS waves are crucial to the acquired adaptation of plant systems (Zandalinas et al., 2020). It was reported that the dependent *D*(*rbohD*) gene inhibits signal propagation by inhibiting ROS accumulation away from the start site, suggesting that ROS waves are RBOHD-dependent (Miller et al., 2009; Mittler and Blumwald, 2015).

The H_2_O_2_ produced by NADPH oxidase acts as a secondary messenger to activate the MAPK, a conserved intracellular pathway in plants consisting of MAP kinase kinase kinase (MAPKKK), MAP kinase kinase (MAPKK), and MAP kinase (MAPK). These kinases are activated step by step through phosphorylation and dephosphorylation. In this process, MAPKKK is first activated; it then activates the downstream MAPKK, and finally, MAPKK will activate the most downstream MAPK, this cascade reaction allows the signal to be amplified and transmitted within the cell (Jonak, 2002). The MAPK cascade pathway is highly correlated with abiotic stresses such as high temperature and drought; for example, the expression level of MAPK was significantly correlated with the relative biomass of plants in response to drought (Liu et al., 2015). Another study showed that *PlMAPK1* plays a positive regulatory role in the plant response to heat stress (Qian et al., 2023). ROS can induce MAPK activation, and interfering with the MAPK cascade can modulate ROS production and response. With further climate change, it is important to investigate the relationships and interaction mechanisms of this pathway with ROS, phytohormones, and other substances in the apoplasts to improve plant resistance to unfavorable conditions. Extracellular peroxidase (ECPOX) is a protein that induces ROS production and detoxification in apoplasts, It is closely related to the formation of extracellular O_2_^•−^ (Li et al., 2010; Gautam et al., 2017).Studies have found that the activity of ECPOX is related to the production of ROS, in the apical cell wall of pea seeds, the activity of ECPOX increases simultaneously with the production of O_2_^•−^ (Kranner et al., 2010).In wheat roots, the production of O_2_^•−^ sensitive to peroxidase inhibitors was taken as evidence indicating that ECPOX was involved in ROS production (Minibayeva et al., 2009).

Polyamines are important regulatory factors of ROS homeostasis (Saha et al., 2015; Kolupaev et al., 2019). H_2_O_2_ in plant cell walls can be produced by the metabolism and recovery of polyamines, of which diamine oxidase (DAO) and polyamine oxidase (PAO)are key to polyamine metabolism (Moschou et al., 2008). PAOs used polyamine as substrate to catalyze the formation of smaller polyamines, as well as a molecule of amino aldehyde and H_2_O_2_. The other was DAO dependent on Cu2^+^ and using pyridoxal phosphate as coenzyme. DAO catalyzes putrescine to produce H_2_O_2_, ammonia and 4-aminobutyraldehyde (Kusano et al., 2007, Kusano et al., 2008). Another characteristic of tomato slpao3 mutant was found to be tolerant to drought stress, indicating polyamine helps to improve drought resistance (D’Incà et al., 2024).

Oxalate oxidase (OXO) in the cell wall catalyzes the oxidation of oxalic acid with molecular oxygen to form CO_2_ and H_2_O_2_ (Verma and Kaur, 2021). Studies have shown that high levels of OXO expression produce a large amount of H_2_O_2_ that also has a direct harmful effect on plants, inducing cell death (Delisle et al., 2001).

### 3.4 ROS in the peroxisomes

Peroxisomes are important sites to produce ROS by oxidative metabolism in plant cells, such as O_2_^•−^, H_2_O_2_, and ^1^O_2_, which participate in different physiological processes and play an important role in maintaining cellular redox homeostasis (Río et al., 2006; Choudhury et al., 2016) (**Figure 3**). Photorespiration has a substantial impact on plant metabolism and is the pathway of ROS production and clearance in peroxisomes (Noctor, 2002; Foyer et al., 2009; Del Río, 2020; Timm and Hagemann, 2020) (**Figure 3**).

Hydroxy pyruvate reductase 1 (HPR1), the main metabolic enzyme in the photorespiration cycle, can be inhibited by high light intensity, which exacerbates the production of ROS (Wang et al., 2022d). It has been found that the ROS metabolism of photorespiration has a dual role, and xanthine oxidase can mediate the production of O_2_^•−^ (Tewari et al., 2009). Xanthine dehydrogenase 1 (XDH1) produces ROS with NADPH oxidase in leaf epidermal cells, and the clearance of H_2_O_2_ in mesophyll cells by XDH1 protects plants from oxidative damage (Ma et al., 2016). Researchers have found that photorespiratory metabolism was downregulated in *Arabidopsis* after hypoxia or the addition of aminooxyacetic acid (a photorespiratory inhibitor), while ROS levels were significantly elevated (Saini, 2023). Another study found that the photorespiration metabolism of rice was upregulated under high temperature and iron stress to ensure survival under stress (De Souza et al., 2024). These studies suggest that the regulation of photorespiration is an important way to maintain ROS homeostasis.

It was found that the H_2_O_2_ content in quinoa increased under high temperature and drought conditions, and Peroxisome abundance was positively correlated with H_2_O_2_ content in the leaves (Hinojosa et al., 2019). The *OsAPX4* gene encoding peroxisomes in rice is thought is thought to affect plant development and resilience by regulating ROS signaling, suggesting that the peroxisome plays a significant role in maintaining ROS homeostasis in plant cells (Ribeiro et al., 2017).

## 4 ROS detoxification

### 4.1 Enzymatic system

Antioxidant enzymes such as CAT, SOD, APX, and glutathione reductase (GR) constitute the ascorbate-glutathione cycle (ASA-GSH cycle), which can effectively scavenge ROS such as H_2_O_2_ in plants. This pathway plays a vital role in helping plants cope with stresses such as high temperature and drought (Tiwari et al., 2017; Del Río, 2020). There are other antioxidant enzymes, such as GPX, glutathione S-transferase (GST), monodehydroascorbate reductase (MDHAR), and dehydroascorbate reductase (DHAR), which together form a complex component of the antioxidant defense system and can effectively scavenge ROS to maintain ROS homeostasis in plants (Zhou et al., 2017, Zhou et al., 2018; Sarker and Oba, 2018; Hasanuzzaman et al., 2019).

SOD can be divided into Fe-SOD, Mn-SOD, and Cu/Zn-SOD isoenzymes according to the different metal co-groups, which can catalyze O_2_^•−^ to H_2_O_2_ (Alscher et al., n.d.). H_2_O_2_ can be further broken down by other antioxidant enzymes such as CAT or APX; CAT can split H_2_O_2_ into water and oxygen, and APX uses ascorbic acid to scavenge H_2_O_2_ under drought stress (Hasanuzzaman et al., 2012; Tiew et al., 2015). A study showed that CAT activity increased significantly under severe drought conditions, while mild and severe drought altered the expression patterns of CAT1 and CAT2. These results suggest that complex regulation at the level of CAT mRNA translation controls the accumulation of H_2_O_2_ in leaves (Luna, 2004). At high temperatures, *SmAPX2* with APX activity can catalyze the oxidation of ascorbic acid by H_2_O_2_ *in vitro* and reduce the accumulation of H_2_O_2_ under high temperature stress *in vivo* through the temporary expression of *SmAPX2*. These results indicate that *SmAPX2* plays an active role in plant responses to high temperature stress (Shen et al., 2024).

The GR-mediated ASA-GSH cycle in the peroxisome has special significance in ROS clearance and tolerance to abiotic stress (Foyer and Noctor, 2011). One study showed that the accumulation of GR gene transcripts under heat stress was 3.1 times that of untreated samples, and the gene was upregulated by various stresses (Chanda Roy and Chowdhary, 2023). *OsNAC092* mutant rice had higher ROS clearance ability and maintained a higher GSH/GSSG ratio and reduction level under drought conditions, thus protecting cells from oxidative stress (Wang et al., 2022a).

A study by explored the response of maize varieties with different heat tolerances to the ASA-GSH cycle under high temperature stress. The results showed that high temperature resulted in increased ROS and increased levels of reduced glutathione (GSH) and oxidized glutathione (GSSG) in all varieties and significantly increased GST activity. The enzyme activities of APX, MDHAR, and DHAR were decreased in sensitive varieties, while the GR enzyme activities were significantly increased in heat-resistant varieties, and the AsA levels were increased in heat-resistant varieties and decreased in sensitive varieties (Tiwari and Yadav, 2020). Another study on wheat under high temperature stress found that both the GR and APX activities increased with increasing heat stress. The GR and APX activities of wheat varieties with higher heat resistance were the highest (Mohi-Ud-Din et al., 2021). Furthermore, when cotton was stimulated by H_2_O_2_, the activity of MDHAR significantly increased. Additionally, SOD, APX and CAT activities also increased significantly (Madhu et al., 2024). These results suggest that *GhMDHAR* can participate in the H_2_O_2_ response and plays an important role in maintaining ROS homeostasis. Recent studies have shown that the *TaMDHAR* gene was significantly upregulated in wheat under drought, high temperature, and high salt conditions. The activity of *TaMDHAR* increased significantly under high temperature and drought stress, while that of MDHAR decreased. It was also found that the MDHAR protein mainly interacted with Asc, DPN, NADP^+^, NADPH, CoA, FAD^+^, MgATP, H_2_O_2_, FMN, MgADP, and GSSG (Madhu et al., 2024). The researchers found that high temperatures increased MDHAR activity in two rice varieties, IR-64(heat sensitivity) and Huanghua Station (heat tolerance), and CAT, SOD, and POD activities were also affected. Moreover, the contents of H_2_O_2_ and MDA in rice were increased under high temperature, and the concentrations of cytokinin and auxin in different plant sites in the tested heat-sensitive and heat-tolerant cultivars were also affected. The study also found that the MDHAR activity was increased following the application of plant growth regulators such as methyl jasmonate and ascorbic acid (Al-Zahrani et al., 2022).

In conclusion, the ASA-GSH cycle plays an important role in plants during stress, and MDHAR has a non-negligible effect on the ASA-GSH cycle and even the antioxidant system of the entire plant. Studies on the ASA-GSH cycle, the enzyme system in the peroxisome, and the regulatory effects of exogenous substances on the ASA-GSH cycle are of great significance for improving crop stress resistance.

### 4.2 Non-enzymatic system

Small-molecule antioxidants exist in plant cells. Some highly toxic ROS, such as ^1^O_2_ and ^•^OH, that cannot be scavenged by the antioxidant enzyme system can neutralize free radicals or produce a relatively harmless free radical by donating electrons or hydrogen atoms by antioxidants, which are later effectively scavenged by other antioxidant systems (Hernández et al., 2009). Antioxidants in plants include AsA, GSH, flavonoids, tocopherols, and carotenoids, among others (Hernández et al., 2009, Hernández et al., 2012; Dangles, 2012; Shen et al., 2018).

Tapetum development and functional defects1 (TDF1) can inhibit tapetum cell division. The downstream target of TDF1, SKS18, was found to encode a polycopper oxidase-like protein with ascorbate oxidase activity. Moreover, TDF1 negatively controls an AsA biosynthetase VTC1 to regulate AsA biosynthesis and maintain appropriate AsA content. SKS18 knockdown or VTC1 overexpression can increase AsA concentration and reduce ROS accumulation, thereby balancing cell division and cell differentiation in the felt layer (Wu et al., 2023b). Flavonol synthetase (FLS) is one of the key enzymes in the synthesis of flavonoids. Under drought stress, overexpression of *EkFLS* in *Arabidopsis* resulted in the increased accumulation of flavonoids and significantly enhanced POD and SOD activities (Wang M. et al., 2021).

Carotenoids and α-tocopherol, as components of the non-enzymatic antioxidant system, can also scavenge ROS. Studies have shown that *NtCCD1* is a negative regulator of carotenoid content in tobacco, and silencing *NtCCD1* by virus-induced gene silencing (VIGS) can increase carotenoid contents and decrease ROS levels (Du et al., 2023). Another study in tomatoes identified a *VTE5* gene that plays a key role in alpha-tocopherol production. When *VTE5* is silenced, the production of α-tocopherol is affected, which reduces the plant’s ability to resist high temperature and light stress. However, the content of α-tocopherol almost doubled under combined stress compared to exposure to high temperatures or high light alone (Spicher et al., 2017).

With the furthering of biological and agronomic research in recent years, the application of exogenous substances for improving the antioxidant capacity and enhancing the stress resistance of plants has become increasingly prevalent. For example, the exogenous application of AsA and α-tocopherol is used to maintain ROS homeostasis and improve drought resistance (El-Beltagi et al., 2022; Zafar et al., 2024). Melatonin was first discovered in the pineal gland of animals, and it is thought to be a free radical scavenger that can be synthesized *in vivo* (Hevia et al., 2014; Vielma et al., 2014; Hardeland, 2017; Zhao et al., 2019). The application of exogenous melatonin in plants has also gained increasing research interest. Studies have shown that exogenous melatonin can effectively reduce the oxidative stress caused by iron deficiency in sweet pepper and enhance antioxidant defense mechanisms. In addition, the chlorophyll content and photosynthetic efficiency of plants under salt stress can be improved (Kaya et al., 2020). Melatonin and hydrogen sulfide regulate the antioxidant defense system under chromium stress, and the supplementation of melatonin to tomato seedlings under stress eliminates excess ROS, thus maintaining ROS homeostasis and improving the stress resistance of tomato seedlings (Khan et al., 2023). Studies on the effects of exogenous melatonin under high temperature and drought stress showed that supplementation with exogenous melatonin could regulate the ASA-GSH system and affect the activity of antioxidant enzymes, thereby protecting plants from oxidative damage caused by drought (Khan, 2023).Other studies have shown that exogenous melatonin can alleviate the negative effects of excess ROS, significantly increase the activity of antioxidant enzymes, stabilize chloroplast structure, and improve the drought resistance of maize seedlings (Muhammad et al., 2023). Moreover, melatonin can enhance the heat resistance of rice seeds by enhancing the activity of antioxidant enzymes and significantly reducing the content of malondialdehyde (Yu et al., 2022). In addition, some studies have shown that the application of exogenous melatonin can increase the activity of antioxidant enzymes and the concentration of metabolites involved in osmoregulation and ion homeostasis in mung bean under drought and high temperature stress, as well as improve the physiological and yield traits of mung bean under combined stress in the reproductive stage (Kuppusamy et al., 2023).

## 5 Nanobiotechnology regulate ROS homeostasis and heat and drought tolerance in plants

### 5.1 Reported nanomaterials maintain ROS homeostasis

Maintaining plant ROS homeostasis can improve plant stress tolerance. With increased research interest in nanotechnology in recent years, the relationship between nanomaterials and plants has attracted increasing attention.

Nanomaterials (NMs) are defined as materials with at least one dimension in the nanoscale (1–100 nm) (Auffan et al., 2009). Due to their small size effect, NMs have unique properties and can penetrate through cell walls and enter plant cells to regulate the complex physiological and biochemical processes of plants, thus regulating plant growth and development. Researchers are taking advantage of the special properties of NMs to study the interaction between NMs and plants (Shi et al., 2014; Ajmal et al., 2023). Researchers alter the properties of NMs by applying different modifications to their surfaces. As a nano-mimetic enzyme, polyacrylic acid-modified manganous tetroxide nanoparticles (PMO) have both SOD and CAT activities, and they can effectively scavenge excess ROS, including H_2_O_2_ (Liu et al., 2023). Studies have shown that under drought stress conditions, PMO can effectively scavenging excess ROS through their nanase activity and alleviate the mitotic activity inhibited by drought by maintaining ROS homeostasis (Sun et al., 2023). Mn_3_O_4_ NPs can enhance the endogenous antioxidant defense ability of plants and relieve the oxidative stress of cucumber plants (Lu et al., 2020). Cerium oxide nanoparticles are also known to be an effective ROS scavenger. The principle of ROS scavenge is to catalyze ROS decomposition by utilizing the oxygen vacancy in their lattice structure (Giraldo et al., 2014). Researchers have found that Poly(acrylic acid) nanoceria (PNC) can scavenge a variety of ROS, including ^•^OH, protect plant photosynthesis, and reduce the damage to plants caused by abiotic stress (Wu et al., 2017). CeO_2_ nanoparticles can also improve the SOD activity and salt tolerance of cotton and rape, Se-NPs scavenge ROS in sorghum under drought stress, relieve oxidative stress, and protect the photosynthesis and grain yield of sorghum (Djanaguiraman et al., 2018b; Liu et al., 2021; Li et al., 2022).

**Figure 4:**
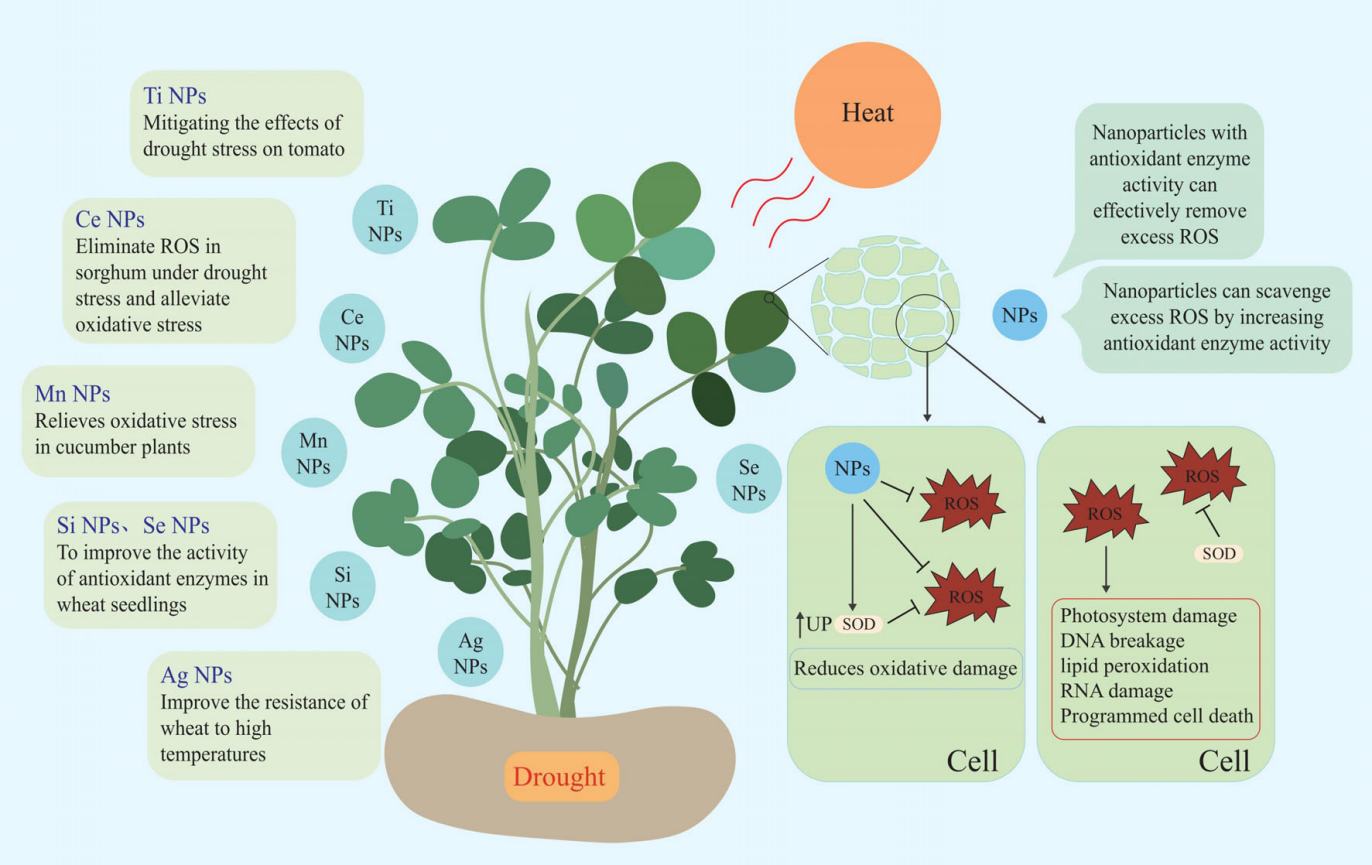
Nanoparticles used to stress tolerance and improve plant growth.

Nanozymes are nanomaterials with enzyme characteristics. Nanoparticles not only directly scavenge ROS but also indirectly improve the ability of plants to scavenge ROS by affecting the activity of antioxidant enzymes (Singh, 2019; Wu et al., 2019). Spraying nano-selenium under drought and high temperature stress increases the antioxidant enzyme activity of wheat seedlings, thereby improving the ability of wheat to resist high temperature and drought stress (Omar et al., 2023). Other nanoparticles such as Ag NPs can improve wheat resistance to high temperatures, while Si NPs help wheat mitigate drought stress, and TiO_2_ NPs alleviate the effects of drought stress on tomato (Iqbal et al., 2019; Boora et al., 2023; Cevik, 2023). Currently, Mn NPs, Ce NPs, Si NPs, Se NPs, Ag NPs, Ti NPs, and other nanoparticles have been applied to wheat, cucumber, rape, sorghum, and other plants to circumvent the negative effects of high temperature and drought stress on plants (Djanaguiraman et al., 2018b; Iqbal et al., 2019; Boora et al., 2023; Cevik, 2023; Omar et al., 2023; Sun et al., 2023; Nauman Khan et al., 2024) (**Figure 4**; **Table 1**).

**Table 1:** Nanoparticles used to stress tolerance and improve plant growth.

Nanomaterials	Mechanisms	Reference
PAA (polyacrylic acid) coated Mn_3_O_4_	scavenge excess ROS, maintaining ROS homeostasis	Liu et al., 2023; Sun et al., 2023
Mn_3_O_4_ NPs	Enhance the endogenous antioxidant defense ability	Lu et al., 2020
Cerium oxide nanoparticles	ROS scavenge is to catalyze ROS decomposition by utilizing the oxygen vacancy in their lattice structure	Giraldo et al., 2014
Poly(acrylic acid) nanoceria (PNC)	Scavenge of ROS and protect plant photosynthesis	Wu et al., 2017
Poly(acrylic acid) nanoceria (PNC)	Maintain cytosolic K^+^/Na^+^	Liu et al., 2021
Poly(acrylic acid) nanoceria (PNC)	Maintain plant cell K^+^ retention	Wu et al., 2018
CeO_2_ nanoparticles	Alleviate membrane oxidative damage	Li et al., 2022
Se-NPs	Enhance the endogenous antioxidant defense ability	Djanaguiraman et al., 2018b
Nano-selenium	Enhance the antioxidant enzyme activity	Omar et al., 2023
Ag NPs	Improving morphological growth.	Iqbal et al., 2019
Si NPs	Scavenge of ROS, increases the antioxidant enzyme activity	Boora et al., 2023
*Salvia Miltiorrhiza-*Derived the synthesized Carbon Dots	scavenge of ROS	Li et al., 2021
Zn NPs	Improve the activity of antioxidant enzymes and drought resistance of wheat	Yang et al., 2018
TiO_2_ NPs	Increase wheat yields	Jaberzadeh et al., 2013
ZnO-NPs	Mitigating the effects of drought stress on sorghum	Dimkpa et al., 2019

Research shows that effects of NMs on plants under stress are diverse. Research shows, that PNC catalytic scavenging of ^•^OH influences the activity of key plasma membrane channels controlling plant cell K^+^ retention, thereby alleviating the damage of salt stress to *Arabidopsis* (Wu et al., 2018). In addition, carbon dots NMs also have ROS scavenging functions (Dong et al., 2022; Innocenzi and Stagi, 2023). *Salvia miltiorrhiza*-derived the synthesized carbon dots have a scavenging effect on intracellular ROS in plants (Wang et al., 2022d) (**Table 1**). Soil application of carbon dots enhanced the N-fixing ability, the expression of genes related to nitrogen transport and water absorption was enhanced of nodules enhanced N and water uptake in soybeans under drought stress, promoted the growth and nutritional quality of soybeans (Wang et al., 2022c).

Recently, researchers synthesized manganese oxide nanoparticles (MnO_2_ MFs) and cerium oxide nanoparticles (CeO_2_ NPs) and regulated the interaction between the two by polydiallyldimethylammonium chloride surface modification, forming Ce−PMn and Mn−PCe composite materials. The formed composites exhibited a variety of enzymatic activities, such as SOD and CAT functions, and the composites Ce−PMn and Mn−PCe retained the nanozymatic activities of individual metal oxides to a certain extent (Alsharif et al., 2023). This suggests that maintaining ROS homeostasis to improve stress resistance may not be limited to a single nanomaterial. The application of composite materials is also an important focus.

In addition to maintaining ROS homeostasis and improving plant stress resistance, the special properties of NMs can also be used as tools for conveying agricultural chemicals, such as nano-fertilizers, and nano-pesticide (Ahmadian et al., 2021; Wang et al., 2022d, Wang et al., 2023; Farajollahi et al., 2023). Based on the special properties of NMs, nano fertilizers also show better utilization efficiency. For example, the application of nano-silica and nano-iron oxide fertilizers under drought stress can improve the activity of antioxidant enzymes and reduce the harmful effect of drought stress on wheat and soybeans, and the application of nano-iron oxide can also increase the activity of soybean nitrogenous enzyme (Ahmadian et al., 2021; Farajollahi et al., 2023). The use of NMs to improve the ability of biological nitrogen fixation not only uses the properties of NMs to target specific organelles for precise and effective delivery but also avoids the risks associated with transgenic methods (Li et al., 2023). In addition to nano fertilizers, nano pesticides show a better control effect than commercial synthetic pesticides in field trials, providing a new and sustainable approach for the control of plant diseases (Wang et al., 2022b). Recent studies have used nanotechnology to design a self-assembled nano bioprotectant based on double-stranded RNA and plant inducers, which enhances endocytosis and amplifies plant system defense responses, effectively helping plants cope with potato late blight infection (Wang et al., 2023).

For a long time, researchers try to investigate the possible mechanisms of nanomaterials enhance plant abiotic stress tolerance. Maintaining ROS homeostasis is the main idea of plant nanobitechology, and the downstream signaling pathway is also being studied. However, few attention has been paid to the material science mechanism.

Nanomedicine has some similarities with plant nanobitechology, and its material science mechanism can be used as reference. Cerium oxide nanoparticles (nanoceria), for instantance, was widely reported could enhance crop abiotic tolerance. Because the surface of NC has two valence states of Ce^3+^ and Ce^4+^, creating an oxygen vacancy (Boghossian et al., 2013), nanoceria can scavenge ROS effectively and enhance the salt resistance ability of plants. Further study on the nanoceria crystal structure shows that the catalytic efficiency of nanoceria with different crystal structures on ROS is different (Wang Z. et al., 2021; Yuan et al., 2023)The functional groups modified on the surface may also affect nanomaterials biological effects (Cheng et al., 2021). But there are no standardized guidelines for agricultural nanomaterials synthesis. Thus, in general, the mechanisms of nanomaterials maintaining plant ROS homeostasis is through Redox reaction from botany, but material science mechanisms need further study.

### 5.2 Issues in nanomaterials and plants interaction

The first step in the interaction between nanomaterials and plants is the cell. The cell wall and membrane are hindrances for nanomaterials entering plant cells. The cell wall has been reported to have pores (Zeng et al., 2017) and negative potential (Fry, 2011). Normally, a particle size smaller than the pore’s diameter and with positive potential can cross the cell wall more easily. Different sizes and potentials affect how nanomaterials enter plant cells. By exploring different leaf structures, researchers have found that they are dependent on a suitable size. However, in both cotton and maize, positive potential nanomaterials have better delivery efficiency. Problems usually have a secondary solution. The cell wall consists of pectin and cellulose; thus, cellulase can damage the cell wall and enable the entry of nanomaterials into the cell (Serag et al., 2012). Groups and ions in nanomaterials are likely to have a decisive influence on the interaction between nanomaterials and plants. Pectin was reported as an key factor for nanomaterials access cell wall (Zhu et al., 2023).

As mentioned above, nanomaterials with a positive potential may have better delivery efficiency, but bio-functions can be quite the opposite. Nanoceria is considered to have a good ROS-scavenging ability but with uncertain bio-functions. Wu et al. (2017) studied different potential ceria bio-functions and indicated that negative potential ceria had the best performance in *Arabidopsis*. This means that the design of nanomaterials also needs to consider their application.

The effects of nanomaterials on plants have been reported with both negative and positive implications. With the development of nanotechnology research interest, too many nanoparticles are emitted into the environment and have imposed a negative impact on plants (Hossain et al., 2015). The way NPs are used need further understanding and determination. Existing application methods, such as foliar sprays and root applications, result in a significant release of nanomaterials to the environment. For food production, a large amount of NPs is absorbed by arable land (Prakash et al., 2021). It is reported that the soil microbial community’s diversity and composition have been altered upon exposure to 100 mg/kg tungsten disulfides nanomaterials (Shi et al., 2022). Another study quantified the movement of NPs through the food chain, starting with NPs adhering to algae, then transferring to Daphnia, and ultimately ending up in fish. The study revealed that NPs were present in fish tissues, with the highest levels found in the brain (Abdolahpur Monikh et al., 2021). It is an important question to study how NPs are applied and how their concentrations affect plants and the environment to create a balance. When NPs are constantly being discharged, concerns about the potential accumulation of nanomaterials in edible parts of crops and subsequent accumulation in the food chain becomes a huge concern, which can pose risks to human health.

Achieving precise delivery of nanomaterials to plants and reducing the impact on the environment is the way forward. Moreover it is essential to establish thorough regulatory frameworks for the utilization of nanomaterials in agriculture and the environment. Creating regulatory guidelines and standardized procedures can help mitigate potential risks and ensure the safe use of nanotechnology for environmental sustainability.

## 6 Conclusion and prospects

The aim of the research and development of plant nanobitechology is to improve plant tolerance to stress and improve crop yield and quality. Murali et al. reviewed the uptake, transport, and bioaccumulation of NMs in plants, as their bioaccumulation has an impact on the food chain via their transportation and accumulation in the edible tissues of plants (Murali et al., 2022). While NMs have the potential to improve agricultural sustainability in crop production, their widespread manufacture and release into the environment are a growing concern. It is necessary to further explore the mechanism by which plant resistance is improved through plant nanobiology and to develop NMs whose production cost is affordable and on par with agricultural products. In addition, it is important that more environmentally friendly NMs are explored to determine the best NMs for application in different crops. The promotion of NMs from the experimental stage to real production applications is also a key issue.
